# Evading the Entrepreneurship: A Study to Discover Implementable Online Approaches to Avoid Greenhouse Consequences

**DOI:** 10.3389/fpsyg.2021.713957

**Published:** 2021-08-09

**Authors:** Lian Feng, Hu Wenting, Tazeem Akhter, Gadah Albasher, Alamzeb Aamir, Asma Imran

**Affiliations:** ^1^School of Philosophy and Social Development, Shandong University, Shandong, China; ^2^Business School, Nanjing University, Nanjing, China; ^3^Business College, Jiangsu Open University, Nanjing, China; ^4^University of Health Science, Lahore, Pakistan; ^5^Department of Zoology, College of Sciences, King Saud University, Riyadh, Saudi Arabia; ^6^Department of Management Science, FATA University, Kohat, Pakistan; ^7^Department of Management Sciences, COMSATS University Islamabad, Lahore Campus, Lahore, Pakistan

**Keywords:** greenhouse gasses, green purchase intention, green entrepreneurship green products, mitigation, e-commerce

## Abstract

Greenhouse gases emissions due to climate change are a continuous threat to the global world, mainly relying on the pervasive consumption of numerous products, including synthetic and non-synthetic products. This research focused on the green purchase intentions of students in Pakistan towards different products, which are related to minimising the greenhouse effect and are available for sale on numerous e-commerce websites, ultimately proceeding to green entrepreneurship. The main objective of this study was to determine which methodology was better among product listing, social media advertising, and online virtual community to enhance customer online green purchase intention while considering online information about the greenhouse effect as a mediating variable. The AMOS 24 was used for this research. SEM was performed with the help of bootstrap methodology. The research was conducted on 280 students at different educational institutes in Pakistan, using a simple random sampling technique. A finding of this study suggested that all three methods positively impacted the green purchase intention of consumers and green entrepreneurship, but online virtual communities could be considered in a more effective way to enhance the green purchase intention of its targeted customers.

## Introduction

Environmental safety is the most critical and emerging concern of society these days. Multiple options and opportunities are being offered for online shopping as the number of base internet users are also increasing day by day (Kabir and Musibau, 2018). Improved and better ecological awareness encourages different companies to offer their goods and services through the internet (Wallington et al., 2009; Huo et al., 2021a). Due to the current scenario caused by COVID-19, the business shifted rapidly towards online shopping. Online shopping has introduced an advanced channel to improve the relationship between the marketer and the customer. The trend of online shopping is increasing rapidly worldwide. All the local and multinational marketers are fighting and competing to attain and retain their valuable customers to achieve their sales targets (Huo et al., 2021a). These technological platforms have enhanced awareness of different available options and made the competition more intense and focused (Jianjun et al., 2021).

An Earth-wide temperature boost because of expanding ozone harming substance (GHG), focusing the environment, has become a significant concern worldwide. Environmental change is shown at a higher rate worldwide, and it is evident through temperatures, rising worldwide mean ocean levels, liquefying ice covers and recurrence of outrageous climate occasions. Most logical examination proposes that the social and monetary outcomes of unabated environmental change could be emotional (Verma, 2015; Öncel and Tzanakis, 2018).

The ascent in GHG fixations is basically because of carbon dioxide (CO_2_) coming from the utilisation of petroleum products, particularly for power age and transport in nations. Another significant wellspring of CO_2_ emanation is deforestation. Along with outflows of methane and nitrous oxides that start principally in the agricultural area, CO_2_ represents almost 99% of worldwide GHG discharges (Karim and Rafi, 2020; Shakoor et al., 2021).

Over the last few years, global consumption has been intensely increasing, ultimately causing a significant and severe environmental disaster. Developed countries consider the dangerous impacts of the consumption of these greenhouse-producing products on their environment and green entrepreneurship (Amen et al., 2021).

Nowadays, the green purchasing is becoming the most important and latest approach for all countries. Green purchasing offers an excellent opportunity to create a barrier to global warming and its continuous serious effects on countries (Li et al., 2020a). That is why the constant effort to save the environment is more appreciated. It is also the need of time to promote and provide information on greenhouse gases emissions so the final consumers could be able to understand their horrible impact on our environment and could move towards those products that are helpful in minimising greenhouse effect (Rodhe, 1990).

Furthermore, customers are more inclined and dedicated to saving and protecting their environment (Sadiq et al., 2020b). For this purpose, they prefer green products instead of buying those products that cause pollution (Huo et al., 2021b).

Green products are becoming a trend and converting into a culture. Previously, anti-greenhouse effect products were not that popular among customers, but, now, they are becoming a sign of luxury life. The interest of the consumers is changing the mindset of the marketer to produce environmentally friendly products (Sadiq et al., 2020b). Customers are more attracted to socially and morally responsible environmentally friendly products (Li et al., 2020b). In this regard, green products are the more popular and leading trend these days, compelling marketers to change and manipulate their strategies (Paustian et al., 2001).

Moreover, a recent study (Joshi and Rahman, 2016) shares that the consumer buying behaviour related to green buying is comprehensively being studied, but it is not an entirely explored concept. In Asian countries, still, there is a great need to share the importance and worth of green products (Dar et al., 2019) because people are still unaware of it, which could be noticed in their buying behaviours. Pakistan is also facing these issues because it is one of the fastest-growing nations. That is why problems are being faced, and different strategies are also offered to cope with the situation. The research question, which is purposed by this study, was to identify the reason behind the attraction of the customers towards a specific product or a brand and to verify whether the information related to greenhouse gases could impact the purchase decision of target customers towards the green products or not. The objectives of the study are given below.

**Objectives of the Study**

1. To study the impact of product listing, social media advertisement and online virtual community on the green purchase intention of the customers while considering the information on greenhouse gases emission as a mediating variable.

2. To identify which technique is more effective and appropriate to enhance the green purchase intention of the target customer positively. So, the customers could purchase those products that help in minimising the greenhouse effect.

3. To recommend the best technique to the organisations so they could be able to implement this specific technique for effective performance and enhanced profitability. Moreover, it could not only enhance the product's sales but also helps in reducing the greenhouse gases emission.

4. To open and identify new avenues for future research so the green purchase intention could be considered as a serious and implementable resolution to save our environment in a broader sense.

### Model Development and Theoretical Support

#### The Commitment Trust Theory of Relationship Marketing

The commitment trust theory of relationship marketing presents a strong background for this study (Morgan and Hunt, 1994). There is a great need to identify the gaps in impacts and significance of greenhouse effects on purchase intentions. For successful relationship marketing, it is imperative to determine which variable has a strong and positive relationship, influencing the purchase decision.

**Figure 1:**
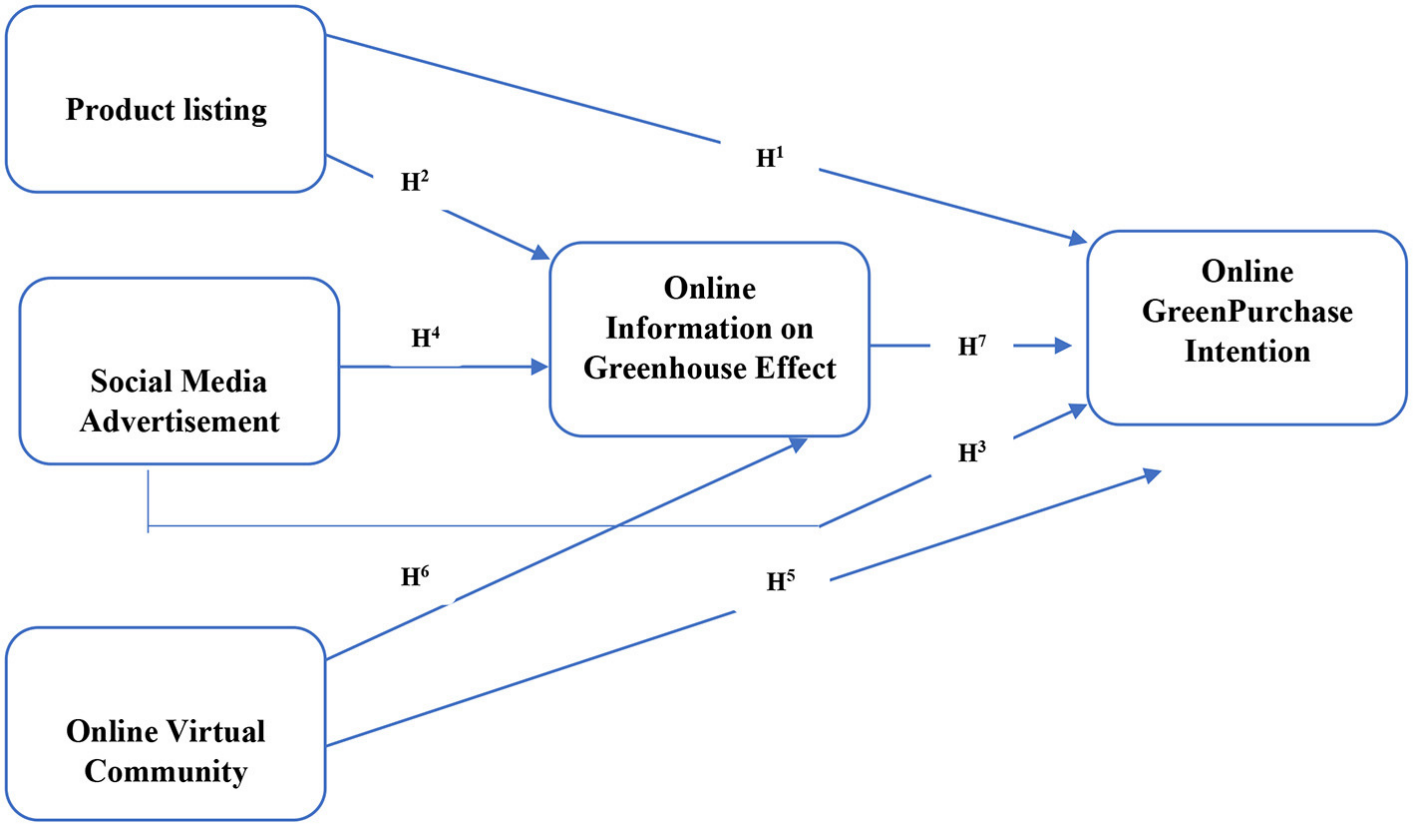
The theoretical framework of potential variable influencing the greenhouse effect.

This theory specifically discusses the consumer relationship with the given product. It is important to identify whether the consumer relates himself or herself with the product successfully or not (Liu et al., 2020). We explored the different variables: online virtual community, online advertisement, product listing with the mediating effect of the greenhouse effect on the buying behaviour of the consumers with a substantial literature background, details given in Figure 1. The study concluded that the most significant relationship between these variables does exist. Firstly, we focused on how these variables work and their importance on the purchase behaviour of the consumers. After that, we targeted those variables that could have the mediating effect on the greenhouse, which increased the worth of even a very casual and straightforward product or brand. Finally, we concluded with the arguments that we need to attach it with some intrinsic or extrinsic characteristics for a successful relationship. As a result, we found that a simple product becomes unique when connected to particular features and is appropriately advertised to address the specific range of customers. It is noticed that online competition is increasing daily. Still, we need to add the additional attributes with our products to exist in the market and compete properly (Jimura and Lee, 2020).

## Literature Review

### Online Virtual Community

Green purchasing is referred to the behaviours or emotions of the customers attached to a single brand due to some specific value addition (Ridings and Gefen, 2004). There are different customer attachment levels and involvement; their dedication to that brand shows their loyalty and behavioural Influence (Ridings and Gefen, 2004). Many of the researchers found that green purchase intention was only attached to the visible attributes of a product. Still, there is a great gap to focus on behaviour or attributes of a firm (Liu et al., 2020). This research focuses on the direct impact of green purchase on the online virtual community as this is our modern market dealing with the all-current issues smartly (Kim et al., 2004). There is a great and direct relationship of green purchase in this online community. After all, they are the ones who can judge and show their behaviour on the spot because they are dealing with the green packaging, green trust, green values, and many more are included, which are directly influencing this community by using this platform (Tian and Guo, 2021).

This study focuses on the relationship between the positive and significant relationship between an online virtual community and online purchase intention, which are greatly affected by the greenhouse effect (Peeters and Pretorius, 2020). Our online community is more concerned (Chandna and Salimath, 2020) about recycling and reuse that is why they promote those brands that are putting up their efforts for the betterment of society (El Morr et al., 2020). Rather than buying from that brand, which is simply fulfilling their needs, they prefer to buy from those who are doing something productive for society. Different studies focus on how their inner satisfaction is attached to their direct reactions (Chou, 2020). There are various pieces of research done to explore the relationship among these variables and check their impact on customer behaviour how they perceive it (Chou, 2020). The further (Chou, 2020) edition of the greenhouse with the purchase intention of the online community will give a clearer perspective on how a customer perceives attributes of a firm while making a purchase decision. Different studies show that different customer demographics demand additional services either for environmental products or just want them best. So, while making any decision for online community customers, we need to focus on their demographic profiles (Chou, 2020).

### Product Listing

Product listing is the heart of online shopping where we need to put all of our efforts to attract and retain our online customers (Ridho, 2021). Use of the green effect while listing our products online could give us a significant boost because it gives a message and a sense of understanding that you are saving your environment by purchasing our product. Moreover, other researchers (Walsh and Dodds, 2017) present that the environment friendly label means the consumer is more attracted to choosing a product that will release environmental stress and make him or her aware of how these products are saving the environment (Purkayastha and Kumar, 2021). Green product listing tries to address all the credible and easily readable information related to environmental safety (Amankwah-Amoah, 2020) states that product listing is an essential and worth considering tool to overcome the pressure of competitors and strengthening the relationship between a product and a customer (Ariffin et al., 2019; Amankwah-Amoah, 2020). Previous studies found that if we add an environment friendly effect to our product listing, it will automatically manipulate the mindsets and behaviour of the consumers (Solekah et al., 2020). They discussed that this thing works as a great marketing tool, but there is a need to share the importance of environment friendly products and their distinct features, which make them superior over regular products (Ezzati, 2021). Therefore, this study determines the impact of a product listing with the addition of information available on the green effect on consumer intention to purchase online, dragged from these findings (Ariffin et al., 2018). Moreover, a significant amount of literature exists, supporting the positive relationship between an impressive product listing and purchase behaviour. But the results and findings of those studies are contradictory from each other (Bebber et al., 2017). The authors found a positive relationship between intrinsic environmental factors, such as perception and actual implementation and their willingness for that behaviour, especially for the certified products in this category (Dangelico and Vocalelli, 2017). The study showed that the consumers with high expectations and great involvement are directly inclined towards the certified products (Wondirad, 2019).

### Social Media Advertisement

Previous studies found that environment friendly ads are included with the three significant elements (Shareef et al., 2019). First of all, an advertisement provides a general statement about the product that how it is environment friendly. After that, an ad itself describes how a company is putting up its efforts to show how and why it is socially and ecologically concerned (Sadiq et al., 2020a). Finally, an ad itself explains the commitment and dedication of the company to the green effect and a wide range of activities related to the campaign and the benefits it is giving to society with its efforts (Hussain et al., 2020). Simultaneously, it was found (Sadiq et al., 2020b) that green ads confirm that the features of a product advertised through online or virtual platforms are more environment friendly. They got appreciation from society as well as raised revenue for their company (Radtke, 2017; Sang et al., 2018). The author made a model that measures the response of a consumer to the green advertisement effect. For example, his or her perception of the brand or product, perception of the typical message through advertisement, his or her behaviour towards the green advertised products and the importance of the message of the ad with his or her routine life (Nguyen et al., 2019).

This variable is the primary and most crucial point of this study, which leads to a specific behaviour derived from the analysis of Rahbar and Wahid (2011) study. As per the definition, it is an activity that may influence behaviour of a consumer by encouraging him or her to purchase those products that are not dangerous for society but will ultimately lead towards the betterment of society by giving positive impacts on the community (Rahmi et al., 2017).

### Information on Greenhouse Effect

For the concept of purchase behaviour, the information available regarding the greenhouse effect works as an iron pillar to manipulate the mind of a customer and encourage him or her for that specific brand rather than the established brand to make a purchase decision of that product to save his or her environment (O'ishi et al., 2009). Some variables need some background or literature support to prove their importance and significance, but the green effect is beyond the word belief (Dar et al., 2019). Many variables support this single concept like motivation, value, attitude, and demographics, which help the purchase intention of the consumers (Cai et al., 2018).

Many of the researchers found that it is simply a desire of one to prioritise his or her needs according to the betterment of the society over the non-green products while making a random or more significant purchase decision (Lee, 2017). The greenhouse effect may influence many of the customers but not all because their choices and demands are different for the fulfilment of the need (Carlson et al., 2017). It is very difficult to predict or forecast the behaviour of a customer that how they will react to a specific campaign of a green effect (Sarkodie and Strezov, 2019). It is imperative to provide valuable information for the mind of the customer that we are giving you the best. The information available on the usage of green products clearly changes the mind of target customers (Meinshausen et al., 2020). When they purchase any product online, then this specific information impacts their purchase decision, and it ultimately forces them to buy those products, which reduce the impact of greenhouse gases.

### Purchase Intention Towards Online Products

It is noticed that online purchase behaviour is getting more normalised and more environment friendly (Sethi et al., 2018) from the context of environment friendly products. We may see a clear difference in a physical store when we need air conditioners and many other facilities that ultimately pollute the environment. On the other hand, online shopping provides a platform where you can buy anything in a routine and comfortable environment (Yu et al., 2018).

As per the theory of planned behaviour by Ajzen and Fishbein (1975), it is found that behaviour is the combination of norms and control or uncontrolled intentions. That is why intention is known as the actual behaviour that cannot be guided or directed. The centre of attention in the Theory of Planned Behaviour intends specific behaviour (Tornikoski and Maalaoui, 2019). Intentions could be controlled by motivation and influenced behaviour. They are a direction towards how people feel and are willing towards how much effort they are putting to show a specific behaviour (Ru et al., 2019). Briefly, it is stated that strong emotion will proceed for actual behaviour, which need to be followed (Ajzen, 2020).

Previous studies found that behaviour of the consumers towards green products is a conscious behaviour effort (Zollo et al., 2018). Their research focused on the intentions of the consumers towards the green products in different prospects. They started from the recycled products and changed them into green products to check the behaviour and intention (Waheed et al., 2020). In similar research of consumer purchase behaviour, the influence of psychological and cultural factors of green products on buying behaviour of consumers is significant (Chen, 2013). The green purchase intention automatically generates or activates the sense of responsibility and makes normal consumers feel special as they contribute to the welfare of society to some extent (Kim and Chung, 2011).

Although (D'Souza et al., 2006) presented a model in which he discussed many variables, which were also related to the green purchase intention and multiple factors affecting the green purchase intention.

The green purchase study deals with the two major variables or dimensions, four Ps, and green purchase intention (Belleau et al., 2007). There might be different factors that may influence this relationship, but we do have those customers who are not ready to compromise even on one factor; they want all in one product with the distinguishing factors like the greenhouse effect, which is eco-friendly and contributing to society within their comfort (Prendergast et al., 2010).

## Research Methodology

A hypothesis research study has been tested for this exploratory study to enlighten the theme of the connexion amid standby variables. The statistics of students of different educational institutes have been drawn from the admission offices of their concerned educational institutes by following the ethical concerns (a certificate attached in supporting information). The reason for selecting students of universities is that they are substantial social media users and have the highest probability of buying products online with the ability to spend money in hand.

This investigational study consisted of a correlational form of examination because it is a prerequisite to patterning the rapport of the variables through hypotheses. The investigation has been directed and gathered in an ordinary atmosphere. This is because it would be contemplated in a non-contrived setting. This study has a nominal investigator meddling with respondents in the matter of satisfying up of questionnaires. In this article, the data are being gathered from students; that is why this examination unit of analysis is discrete. The researcher has instrumented a cross-sectional scheme for this study. It contains the information of all a population, or an illustrative subset, at one precise point at the same time.

### Empirical Settings and Data Collection

Students of numerous universities and educational institutes of Punjab province (Vehari, Sahiwal, Lahore, and Multan) were considered as respondents in this research. Almost 500 questionnaires were distributed among them, and nearly 280 surveys had been acknowledged, upholding the answer rate of 55%. A reliable and valid questionnaire were used for this research. Among these, 30 questionnaires were with incomplete data, so the analysis was carried out with 250 complete responses. The demographic profile of the respondents is discussed in Table 1.

**Table 1:** Demographic profiles of the respondents.

**Category**	**Subdivision**	**Frequency**	**Percentage**
Marital status	Married	150	60
	Un-married	100	40
Age	Below 25 years	50	2
	25–30	85	34
	31–35	65	26
	36–40	40	16
	40 and above	10	4
Education	Intermediate	110	44
	Bachelors	128	51
	Masters	12	5
	M. Phil	0	0
	Ph.D.	0	0
Internet usage	Once in a day	202	80.5
frequency	After 3 days	17	6.8
	After 1 week	6	2.4
	After 2 weeks	2	8
	After 1 month	24	9.6

The respondents were students from a diversity of colleges and universities and were chosen accidentally, using a simple random sampling technique. The respondents had to have experience using social media or online purchasing websites so that they could answer the questionnaire with more information and awareness.

### Measure and Methods

#### Instrument

For measuring Online Virtual Community, the scale is used, which is developed by Kim et al. (2004), and it uses Likert scale, ranging from strongly agree to strongly disagree; it is slightly modified and adjusted while keeping in mind the requirements of the study and further verified by the CFA analysis. To measure the concept of a product listing, we used the scale developed by Adnan (2014). While, for measuring social media advertisement, we used a scale that was developed by Logan et al. (2012), and, for measuring the greenhouse effect, we used the scale that was developed by Staats et al. (1996); it was slightly modified by the authors to fit the scope of this study and then verified by the CFA methodology. Finally, Duffett developed the online purchase in 2015 (Duffett, 2015), which was also modified according to the requirements of this study, and it was also verified with the CFA methodology. The instruments were rated and portioned on a five-point Likert scale with advanced numerical standards, showing greater fulfilment.

#### Confirmatory Factor Analysis

It is essential to comport the confirmatory factor analysis for exact and detailed outcomes for all constructs. For this exploration, it is categorical to check and determine a pooled analysis of CFA. It performs all the latent constructs at the identical period to realise the necessitated model fitness. The pooled CFA technique is a lot calmer and improved than the separate CFA since it runs all the latent variables concurrently, which is a good technique of time-saving (Afthanorhan et al., 2014; Chong et al., 2014). The model fit indices display a suitable fit between the data and the planned measurement model. The charges and figures of the comparative fit index (CFI =0.938), root mean error of approximation (RMSEA = 0.049), and Chi-square to degree of freedom ratio (× 2/df = 1.590) is all meeting the cut-off criteria, so the charges of the fitness indices meet the outstanding standards for model fitness; details are given in Tables 2, 3 (Lomax and Schumacker, 2004; Hoe, 2008; Anderson et al., 2010).

**Table 2:** Pooled confirmatory analysis model fitness tests.

**Name of category**	**Name of index**	**Index full name**	**Value in analysis**	**Acceptable value**	**Literature**
Absolute fit	RMSEA	Root mean square of error approximation	0.059	<0.80	Browne and Cudeck, 1993
Incremental fit	CFI	Comparative fit index	0.948	>0.90	Bentler, 1990
Parsimonious fit	Chisq/df	Chi square/Degrees of freedom	2.590	<3	Hu and Bentler, 1999

**Table 3:** Pooled confirmatory factor analysis (independent, mediating, and dependent variable).

**Scale**	**Items**	**Factor loadings**	**Scale reliability**
Product listing	I buy from online stores only if they are visually appealing and have a well-organised appearance	0.739	0.719
	I buy from online stores only if the navigation flow is user friendly	0.740	
	I buy from online stores only if the site content is easy for me to understand and the information provided is relevant	0.656	
	I buy from online stores only if they have an easy and error free ordering and transaction procedure	0.742	
Social media advertisement	Social media advertising is a good source of product information and supplies relevant product information	0.770	0.777
	Social media advertising provides timely information	0.993	
	Social media advertising is a good source of up-to-date product information	0.558	
	Social media advertising is a convenient source of product information	0.856	
	Social media advertising supplies complete product information	0.708	
Online virtual community	The use of bulletin boards and e-mail exchange among community members affects my decision-making skills	0.707	0.705
	Availability of operational suggestions by community members affects my decision-making skills	0.739	
	Membership creates a sense of belongings in myself towards my community	0.640	
	The opportunity to suggest ideas to the community by one member affects other community members' decision-making	0.701	
	The similarity of the member's interest creates a sense of belongingness within a community	0.776	
	Availability of useful information related to different product and services from community members helps me to take the right decision in my choices	0.656	
	The level of satisfaction with the community's information affects my decision-making toward any product or services available online	0.689	
	I feel that future participation in community activities must change my thinking process in the longer run	0.735	
Information on greenhouse effect	I will not buy those products which causes Greenhouse effect and due to which the earth becomes warmer	0.742	0.741
	I will not buy those products which causes Greenhouse effect and due to which the floods become more likely	0.802	
	I will not buy those products which causes Greenhouse effect and due to which the deserts will appear	0.816	
	I will not buy those products which causes Greenhouse effect and due to which the production of carbon dioxide increases	0.605	
Online green purchase	I will purchase products that are publicised on social media and prevents greenhouse effect	0.825	0.709
intention	I desire to buy products that are endorsed on advertisements on social media and prevents greenhouse effect	0.763	
	I am likely to buy products that are encouraged on social media and prevents greenhouse effect	0.543	
	I plan to purchase products that are promoted on social media and prevents greenhouse effect	0.705	

After successive execution of the pooled CFA, it is also required to pattern and confirm reliability of each item for additional research. CFA of data of the current study was practiced to measure reliability, discriminant validity, and the convergent validity as well. The reliability of the measurement scales was being measured with the composite reliability, which is favoured to report reliability of a scale (Netemeyer et al., 2003), an extensively used indicator.

The discriminant validity is being used to approve that the measurement scales are separate from other measures practised in the study. Discriminant validity was measured by means of the HTMT analysis in which the cut-off trend of strict discriminant validity was 0.850 and, for liberal discriminant validity, was 0.900 (Henseler et al., 2015). Therefore, it is found that all the measurement scales being taken into consideration fluctuate from each other, so the data analysed and presented in our study accomplished the obligations of convergent and discriminant validity and were appropriate for additional analysis; detail is given in Tables 4, 5.

**Table 4:** HTMT analysis of variables.

	**Product listing**	**Social media advertisement**	**Online virtual community**	**Greenhouse effect**	**Green purchase intention**
Product listing					
Social media advertisement	0.275				
Online virtual community	0.272	0.167			
Greenhouse effect	0.107	0.095	0.050		
Green purchase intention	0.320	0.070	0.055	0.578	

**Table 5:** Structural equation modelling or model fitness test.

**Name of Category**	**Name of index**	**Index full name**	**Value in analysis**	**Acceptable value**	**Literature**
Absolute fit	RMSEA	Root mean square of error approximation	0.047	<0.80	Browne and Cudeck, 1993
Incremental fit	CFI	Comparative fit index	0.715	>0.90	Bentler, 1990
Parsimonious fit	Chisq/df	Chi square /Degrees of freedom	1.919	<3	Hu and Bentler, 1999

#### Structural Equation Modelling

Structural equation modelling (SEM) was used in the structural model to test the hypotheses, using AMOS 24. As the proposed model contains mediation, the SEM technique was used to analyse all of the paths simultaneously (Iacobucci et al., 2007; Hoe, 2008; Alavifar et al., 2012). The model fit indices for the structural model are meeting the acceptance criteria.

#### Results From Hypothesis Testing

The SEM statistics show that H^1^ (product listing green purchase intention), H^3^ (social media advertisement green purchase intention), and H^5^ (online virtual community green purchase intention) were rejected on the grounds of a significance level, as the SEM results show that the *P*-values of these hypotheses are not significant. These results suggest that these variables do not have a direct significant positive impact on green purchase intention of the target customers. While H^7^ (greenhouse effect green purchase intention) was accepted on the grounds of a significance level as the SEM results show that the *P*-values of these hypotheses were significant. These results suggest that this variable has a direct significant positive impact on green purchase intention of the target customers. Moreover, the results also indicated that information related to the greenhouse effect could lead to positive green purchase intention.

The results of the structural model are shown in the following Tables 6, 7.

**Table 6:** Results of the structural model: direct effects (H^1^, H^3^, H^5^, and H^7^).

**Hypothesis**	**Causal path**	**Lower bound**	**Upper bound**	***P*-Value**	**Standardised estimated**
H^1^	Product listing → Green purchase intention	−0.162	0.093	0.790	−0.032
H^3^	Social media advertisement → Green purchase Intention	−0.183	0.026	0.306	−0.080
H^5^	Online virtual community → Green purchase intention	0.096	0.378	0.605	0.335
H^7^	Greenhouse effect → Green purchase intention	0.219	0.464	0.003	0.430

**Table 7:** Results of the structural model: direct effects (H^2^, H^4^, and H^6^).

**Hypothesis**	**Causal path**	**Lower bound**	**Upper bound**	***P*-Value**	**Standardised estimated**
H^2^	Product listing → Greenhouse effect → Green purchase intention	0.060	0.174	0.005	0.20
H^4^	Social media advertisement → Greenhouse effect → Green purchase intention	0.027	0.140	0.045	0.25
H^6^	Online virtual community → Greenhouse effect → Green purchase intention	0.052	0.153	0.026	0.45

These results showed the complete picture of this research study. The study showed that **H**^**2**^ **(product listing information on greenhouse effect green purchase intention**, **β** **=** **0.20**, ***P* =** **0.005)** is positively significant and suggests that when websites impressively use the listing factor of a product, then it is effective in enhancing the target customer green purchase intention online. But it is only possible when it is mediated by the information available regarding the impact of greenhouse gases on our society and culture collectively.

The study showed that **H**^**4**^ **(social media advertisement information on greenhouse effect green purchase intention**, **β** **=0.25, P** **=0.045)** is also positively significant and suggests that organisations using a social media advertisement to promote their products online are successful in creating a positive impact on online green purchase intention of their targeted customers. But it is only possible when it is mediated by the information available regarding the impact of greenhouse gases on our society and culture collectively.

This hypothesis showed that **H**^**6**^ **(online virtual communitiesperceived risks online purchase intention**, **β** **=** **0.45**, ***P* =** **0.026)** is positively significant and suggests that online virtual communities could play their role to impact the purchase intention of their targeted customers towards greenhouse impactable products. But it is only possible when it is mediated by the information available regarding greenhouse impact.

## Discussion

Our findings suggested that the relationship of all three variables, which include a product listing, social media advertisement, and online virtual communities, is well-connected with the green purchase intention of targeted consumers. As suggested by different researchers, most environmental behaviour studies utilised reasoned action and planned behaviour theories (Ajzen and Fishbein, 1975) to suggest pro-environmental attitudes directly or indirectly (Dunlap et al., 2000). We found that pro-environmental attitudes were significantly associated with well-marketed products, but only when they are mediated by the information related to the greenhouse effect available online.

These activities are typically practical and promote social status. Because promotion and awareness only encourage publicly visible environmentally friendly behaviours (Addo et al., 2020), our findings suggested that policymakers in different organisations currently providing products related to the greenhouse effect should develop to make environmental actions more noticeable, thereby promoting visible and acceptable social status. Making information available to greenhouse effect products would achieve organisational and consumer goals (Ali et al., 2021). Indeed, simply providing a good level of knowledge in product listing pages could instigate greenhouse product usages in different householders up to a significant level (Amen et al., 2021). Moreover, similar or even more powerful impacts could be achieved through social media advertisements and online virtual communities. However, these exertions to encourage expressive environmental performances must compete with enormous marketing energies to capitalise on green rewards for diverse products also called greenwashing. A dissimilarity between greenwashing where doubtful claims are made and where green marketing, where the shares are more precise, could be ended. But, in repetition, very few inventions promoted in either way have evocative environmental impressions relative to greenhouse effect product usage (Ramus and Montiel, 2005). Although training has originated that green consumerism has slight or no properties on plummeting the environmental influences of consumption (Li et al., 2020b), most consumers may obtain their knowledge about green products from green marketing that aims to capture the green purchase intentions of the consumers (Sadiq et al., 2020b; Abdullah et al., 2021a).

Education provided more evidence for the connection between green consumerism and more meaningful environmental behaviours. Education has long been associated with pro-environmental attitudes and behaviours (Huo et al., 2021a), and both were related to green consumerism. Recent research on the higher level of education has suggested more pro-environmental attitudes and behaviour towards greenhouse effect products (Jianjun et al., 2021).

### Theoretical Implications

As the study concludes, it is imperative to find out its theoretical implications to make it more coherent and understandable from a theoretical perspective. This study used the commitment trust theory of relationship marketing and theory of planned behaviour as the base theory for this research. This proposed theoretical model and the result of this study clearly show that the study successfully justifies the basic concept of both above-described theories, and it is also used and executed in previous researchers (Carattini et al., 2015; Raybould et al., 2020; Huo et al., 2021a).

### Managerial Implications

The main objective of the research study is to provide implementable and workable solutions to the organisations and managers so they could be able to achieve benefits from that specific research study. Our results suggested that online virtual communities could play a vital role in developing a positive purchase intention in our targeted consumers when they are mediated by the online information related to the effects of the greenhouse on our society and culture.

Moreover, the product listing and social media advertisement are also effective and valid but have minimal impact as compared to the online virtual communities (Chan et al., 2020; Li et al., 2021). So, this study will recommend managers of the different organisations, which are working with the selling of green products. They must need to focus on the development of online virtual communities for the promotion of their products because the greenhouse gases are the phenomena that need extensive information and target customers are unlikely to buy these products unless they have a strong and justifiable support from a learned society (Sridhar Balasubramanian, 2001; Sohn and Leckenby, 2007). Using the online virtual communities could bring more effective results and performance as compared with a product listing and a social media advertisement, which also claim a huge chunk of organisational resources for promotion of their green products (Calixto et al., 2017; Alalwan, 2018).

### Future Research Directions

Future research could focus on the new avenues of product information that is available online. But they must also need to focus on the demographics and geographic limitations, which could play a vital role in changing the results of this kind of study (Abdullah et al., 2021b). It is also recommended to conduct this research in different or even the same geographic locality with the same demographically available respondents for more reliable and robust results. So, the organisations could be able to take more appropriate and informed managerial decisions about their greenhouse products.

## Conclusion

As we know, information related to the effect of greenhouse impact on our lives and society is minimal when we consider the knowledge base of our target customers. Organisations that provide these information details are not using a practical methodology to promote their products. Moreover, Pakistan is a country where people do not have that much information related to the greenhouse effect.

That is why we could see that, although all three methods have a positive impact on the green purchase intention of the target customer. Although, the effect is mediated by the online information available on greenhouse impact. But the most successful methodology is online virtual communities in this segment. The reason behind that relation is that target customers are more knowledgeable about any product and services in online virtual communities than the information available on the product listing or in any social media advertisement. Members of the community could ask other members about their experiences with specific products and services and use their knowledge or wisdom while purchasing any expensive product or services related to that matter. These communities generate more confidence in the target customer towards these products, resulting in a more positive green purchase intention.

### Limitations of the Study

Time and resource could be considered as a serious limitation for this study. Moreover, it must be considered in mind while conducting any future research that these results are specifically related to the above-described respondents. They must be verified in other demographic, psychographic, and geographic localities as well to make it more authentic and reliable. It is also possible that results could vary when collecting that same data from the same population because numerous other variables, which could not be considered at that specific time in the study, could play their significant role in mindsets of the respondents. So recurrence of the study is needed to reach more authentic and reliable results for more sustainable and workable corporate solutions in the future.

## Data Availability Statement

The original contributions presented in the study are included in the article/supplementary material, further inquiries can be directed to the corresponding author/s.

## Ethics Statement

The data was collected with the consent of respondent, and ethical approval was taken from University of Health science Lahore, Pakistan.

## Author Contributions

LF and HW: initial draft and supervised the draft. TA and GA: data analysis and improvement of draft. AA and AI: data interpretation.

## Conflict of Interest

The authors declare that the research was conducted in the absence of any commercial or financial relationships that could be construed as a potential conflict of interest.

## Publisher's Note

All claims expressed in this article are solely those of the authors and do not necessarily represent those of their affiliated organizations, or those of the publisher, the editors and the reviewers. Any product that may be evaluated in this article, or claim that may be made by its manufacturer, is not guaranteed or endorsed by the publisher.
